# Evaluation of Ovarian Stromal Microvascularity and Clinical-Hormonal Associations in Reproductive-Aged Women with Polycystic Ovary Morphology

**DOI:** 10.3390/diagnostics15111376

**Published:** 2025-05-29

**Authors:** Hakan Baş, Süleyman Filiz

**Affiliations:** 1 Department of Radiology, Gazi Mustafa Kemal Occupational and Environmental Diseases Hospital, Silahtar Street No:6, Emniyet, Yenimahalle, Ankara 06560, Türkiye; suleymanfilizz@gmail.com; 2 Department of Radiology, Faculty of Medicine, Ufuk University, Mevlana Boulevard No:86–88, Balgat Campus, Ankara 06520, Türkiye

**Keywords:** polycystic ovary syndrome, microvascular imaging, ultrasound imaging, hyperandrogenism, quantitative analysing

## Abstract

**Background/Objectives:** This study aims to assess ovarian stromal vascularity using microvascular imaging in reproductive-aged women with polycystic ovarian morphology (PCOM) and to explore its associations with endocrine parameters and polycystic ovary syndrome (PCOS) phenotypes. **Methods:** We conducted a retrospective, single-center study between January 2021 and November 2023. Women aged 18–49 who met the PCOM criteria (≥20 follicles measuring 2–9 mm or an ovarian volume >10 cm^3^ in at least one ovary) were included. Pelvic ultrasound with MV-Flow Doppler imaging was used to quantify the stromal vascularity index (VI). On the same day, serum levels of FSH, LH, total and free testosterone, DHEAS, and estradiol were measured. PCOS phenotypes (A, C, D, and non-PCOS) were classified according to the Rotterdam criteria. Statistical analysis involved interobserver agreement using intraclass correlation coefficients (ICCs), correlation analysis for hormonal associations, and group comparisons using ANOVA. **Results:** A total of 111 women (mean age: 27.4 ± 6.1 years) were evaluated. The mean VI was 43.88 ± 19.84, with good interobserver agreement (ICC = 0.79; 95% CI: 0.65–0.88). VI was highest in Phenotype A (61.36 ± 10.11), followed by Phenotype C (42.57 ± 3.59), Phenotype D (26.47 ± 4.24), and Non-PCOS individuals (9.95 ± 5.44; *p* < 0.001). VI showed strong positive correlations with total testosterone (r = 0.797) and free testosterone (r = 0.778), and a moderate negative correlation with DHEAS (r = −0.483; *p* < 0.001). **Conclusions:** Microvascular imaging is a promising tool for quantifying ovarian stromal vascularity in PCOM. Its strong correlation with androgen levels, especially in hyperandrogenic phenotypes, highlights its potential role in enhancing diagnostic precision and deepening our understanding of PCOS pathophysiology.

## 1. Introduction

Polycystic ovary syndrome (PCOS) is a common endocrine disorder, affecting approximately 5–10% of women of reproductive age worldwide. It is a leading cause of anovulatory infertility and is frequently associated with various metabolic disturbances [1,2,3]. PCOS is defined by irregular ovulation, hyperandrogenism, and the presence of multiple ovarian follicles, which together contribute to a broad spectrum of clinical presentations that complicate both diagnosis and management [3]. Despite extensive research, the precise etiology of PCOS remains unclear, reflecting a complex interplay between genetic, endocrine, and metabolic factors [3,4]. In 2003, the Rotterdam criteria were established for PCOS diagnosis, requiring the presence of at least two out of three features: oligo/anovulation, clinical or biochemical hyperandrogenism, and polycystic ovarian morphology (PCOM) on ultrasound [3]. While these criteria improved diagnostic sensitivity, they also introduced greater clinical heterogeneity. Consequently, PCOS is now recognized as a syndrome encompassing several phenotypic variants, primarily based on the combination of PCOM, hyperandrogenism, and ovulatory dysfunction [5]. According to the Rotterdam classification, four distinct phenotypes of PCOS have been described. Phenotype A is the “classic” form and includes all three diagnostic features: hyperandrogenism, ovulatory dysfunction, and polycystic ovarian morphology (PCOM). Phenotype B includes hyperandrogenism and ovulatory dysfunction but lacks PCOM. Phenotype C includes hyperandrogenism and PCOM with preserved ovulation. Phenotype D includes ovulatory dysfunction and PCOM without hyperandrogenism. These phenotypes vary in terms of clinical severity and metabolic risk profiles, and distinguishing between them is crucial for appropriate diagnosis and management [6]. Polycystic ovarian morphology (PCOM), a key diagnostic criterion for PCOS, is also observed in 18–25% of otherwise healthy women. This overlap raises concerns regarding the specificity of current diagnostic approaches and underscores the need for additional criteria to improve diagnostic precision. [7,8]

Hyperandrogenism, another hallmark feature of PCOS, may manifest clinically and/or biochemically [9,10]. This androgen excess primarily results from dysregulated theca cell steroidogenesis, increased luteinizing hormone (LH) secretion, and heightened theca cell responsiveness [11,12]. The hyperandrogenic state disrupts normal folliculogenesis, leading to follicular arrest, anovulation, and menstrual irregularities [13,14].

Understanding ovarian blood supply is essential to elucidate the mechanisms underlying PCOS. Research has demonstrated that women with PCOS, particularly those with hyperandrogenic features, exhibit increased blood flow in the ovarian stroma. This atypical vascularization is linked to angiogenesis driven by hyperandrogenism, changes in vascular endothelial growth factor (VEGF) expression, and persistent inflammation. The resulting neovascularization enhances stromal perfusion, which may further exacerbate hormonal and reproductive disturbances. This vascular phenotype is clinically relevant for interpreting ultrasound findings, guiding treatment strategies, and monitoring disease progression. Recent advancements in microvascular imaging (MVI) technology have improved the detection and analysis of changes in ovarian stromal vessels, opening new research avenues regarding the association between irregular blood flow and PCOS development. Investigating ovarian stromal vascular indices alongside hormonal parameters may yield insights into the mechanisms, progression, and potential therapeutic targets of PCOS [15,16,17,18,19].

This study seeks to elucidate the association between advanced Doppler-based assessments of ovarian microvasculature and hormonal profiles in women of reproductive age exhibiting PCOM.

## 2. Materials and Methods

### 2.1. Study Design

This retrospective single-center, study was conducted in accordance with the Declaration of Helsinki and was approved by the Clinical Research Ethics Committee of Ankara Medipol University (Approval Number: 18, Date: 12 February 2024). All patients provided written informed consent for the use of their data in this study. No animal studies were performed. All data were collected from the hospital’s Radiology Department records between January 2021 and November 2023. During this interval, consecutive adult female patients scheduled for routine suprapubic pelvic ultrasonography were identified, and relevant clinical, imaging, and laboratory data were retrieved from the institutional electronic database.

### 2.2. Study Population

The study retrospectively enrolled women of reproductive age (≥18 years) who underwent routine suprapubic pelvic ultrasonography in our institution between days 3 and 7 of their menstrual cycle.

#### 2.2.1. Inclusion Criteria

- Age ≥18 years (up to 49 years) at the time of imaging.
- Polycystic ovarian morphology (PCOM) on ultrasound, defined according to the most recent international guidelines as ≥20 follicles (2–9 mm in diameter) and/or ovarian volume >10 cm^3^ in at least one ovary [3].
- Availability of a complete hormonal profile (FSH, LH, total testosterone, free testosterone, DHEAS, estradiol), obtained on the same calendar day as the ultrasonographic assessment (specifically on menstrual cycle days 3–7, if applicable).

#### 2.2.2. Exclusion Criteria

- Incomplete or missing hormonal data on the day of ultrasound.
- Recent use of hormonal medications (within six months) for menstrual regulation, contraception, or management of PCOS, as these may alter endogenous hormone levels and vascular measurements.
- Any clinical or ultrasound finding (e.g., large ovarian cysts, ovarian masses) that could confound measurement of the ovarian stroma or its vasculature.
- Inadequate image quality (e.g., absent or incomplete cine-loop recordings) precluding a precise vascularity index (VI) evaluation.

### 2.3. Imaging Protocol and Data Collection

Patients were enrolled consecutively sampling approach, and ultrasonographic evaluations were performed during routine clinical practice. Initially, assessments were carried out by one of two experienced radiologists (with 10 and 7 years of expertise, respectively), based solely on patient assignment at presentation, as part of standard clinical care. Consequently, radiologists accessed clinical and laboratory data during these initial evaluations. All patients underwent examinations in the supine position with a full bladder. Images were captured using gray-scale and MV-Flow Doppler imaging and securely stored locally after anonymization by removing all identifying DICOM tags. Subsequently, anonymized images meeting quality criteria—specifically, those acquired in the longitudinal plane with cine-loop recordings of at least 3 s duration—were independently re-evaluated by both radiologists, who remained blinded to clinical information and laboratory results, to determine interobserver agreement for vascularity index measurements. Images failing to meet these quality criteria were excluded from the analysis.

- Gray-Scale Ultrasonography (USG): A Samsung RS85 Prestige Ultrasound System (Samsung Medison) with a convex transducer (CA1-7A, 1–7 MHz) was utilized to perform gray-scale ultrasonographic assessment. The evaluation of each ovary involved a systematic examination for polycystic ovarian morphology (PCOM). This condition was characterized by either the presence of 20 or more follicles with diameters ranging from 2 to 9 mm, or an ovarian volume greater than 10 cm^3^.
- Doppler Examination (MV-Flow, MVI): Ovarian stromal vascularity was evaluated using the same ultrasound equipment and transducer (Samsung RS85 Prestige with CA1-7A, 1–7 MHz). MV-Flow Doppler imaging was performed consistently in the longitudinal plane to maintain anatomical standardization. Cine-loop clips of at least 3 **s** duration were captured and stored. For vascularity index measurements, the optimal frame demonstrating clear vascular flow was selected from the stored cine-loop clips. In the ovarian stroma, three circular regions of interest (ROIs) were centrally positioned. These ROIs, each with a fixed 5 mm diameter, were carefully selected to exclude dominant follicles and peripheral vascular structures. The ROI dimensions and MV-Flow Doppler imaging parameters, including pulse repetition frequency (PRF), gain, and wall filter, were maintained consistently throughout the duration of the study.
- Hormonal Parameters: The acquisition of hormonal measurements was facilitated through the retrieval of data from the hospital’s electronic repository. The extracted parameters encompassed a comprehensive array of endocrine markers, including FSH, LH, total testosterone, free testosterone, DHEAS, and estradiol. All hormonal samples were collected on the same day as the ultrasonographic evaluation, specifically between days 3 and 7 of the menstrual cycle, in the morning hours, to minimize variability and ensure temporal consistency between imaging and biochemical assessments.
- Index Test and Reference Standard: The index test in this study was advanced microvascular Doppler imaging (MV-Flow), used to quantify ovarian stromal vascularity. The reference standards included an ultrasound-based assessment of ovarian morphology to identify polycystic changes and a biochemical analysis of hormonal levels reflecting hyperandrogenism and gonadotropin status. These hormonal assays were used to characterize the endocrine profiles of the patients and to classify polycystic ovary syndrome (PCOS) phenotypes.
- Reading Strategy and Observer Blinding: Initial imaging was performed by one of two radiologists with 10 and 7 years of experience, respectively, during routine clinical care, with partial access to clinical and laboratory data in accordance with standard workflow. For study purposes, all cine-loops and images were anonymized by removing DICOM identifiers, and the same two radiologists independently re-evaluated the stored cine-loops to measure the vascularity index (VI) while being blinded to clinical and biochemical results.

### 2.4. Statistical Analysis

All data were de-identified, and analyzed using SPSS (Version 23.0). The Kolmogorov–Smirnov test was employed to assess normality for continuous variables; those displaying a normal distribution were presented as mean ± standard deviation, whereas non-normally distributed variables were reported as median (interquartile range). Interobserver agreement between the two radiologists for VI measurements was evaluated via the interclass correlation coefficient (ICC), with thresholds set as follows: <0.50 (poor), 0.50–0.75 (moderate), 0.75–0.90 (good), and >0.90 (excellent). For multiple-group comparisons, analysis of variance (ANOVA) or the Kruskal–Wallis test was utilized, followed by suitable post hoc tests when indicated. Pairwise comparisons were conducted using either the independent-samples t-test for normally distributed data or the Mann–Whitney U test for non-normally distributed data. Correlations between VI and hormonal parameters (FSH, LH, total/free testosterone, DHEAS, estradiol) were explored using Pearson’s or Spearman’s correlation coefficients, depending on distributional characteristics. A *p*-value of <0.05 (two-tailed) was considered statistically significant.

## 3. Results

**Figure 1:**
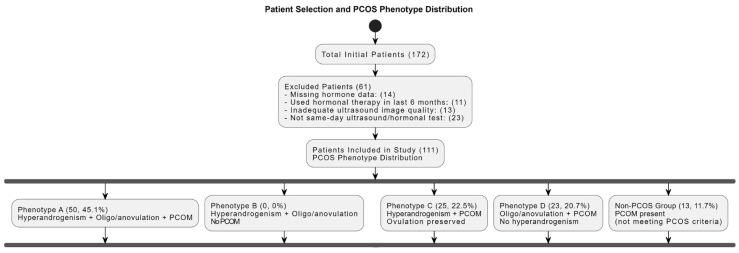
Patient selection and PCOS phenotype distribution. Flowchart summarizing the inclusion/exclusion criteria and classification of patients based on polycystic ovarian morphology and associated endocrine profiles. PCOM = polycystic ovarian morphology; PCOS = polycystic ovary syndrome.

The study encompassed 111 subjects aged 18–42, with a median age of 23 (21–26). Patient selection criteria and PCOS phenotype distribution are presented in Figure 1.

**Figure 2:**
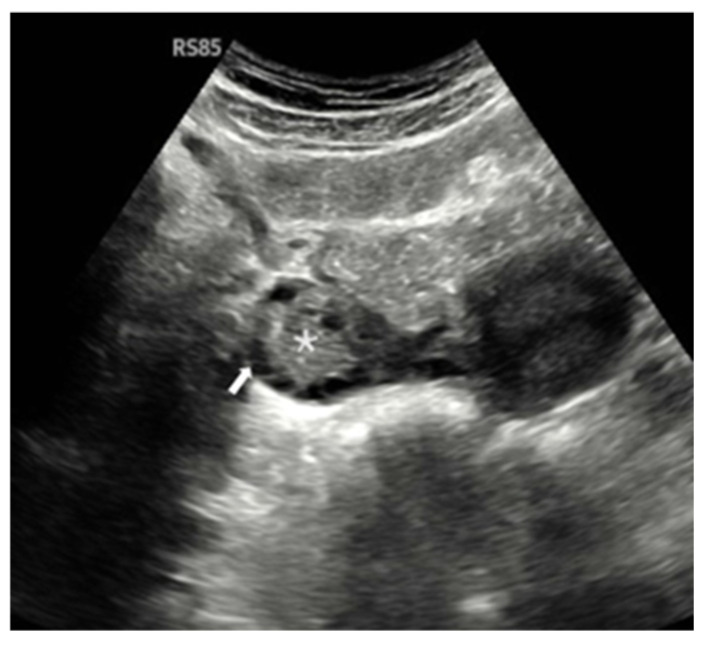
Gray-scale transabdominal ultrasound image in a 22-year-old female demonstrating polycystic ovarian morphology (PCOM). The central ovarian stroma is marked by an asterisk (*), and multiple peripheral follicles (each measuring 2–9 mm in diameter) are indicated by the white arrow. The ovarian volume was calculated as 12 mL. The presence of a prominent central stroma, numerous peripherally arranged follicles, and increased ovarian volume are consistent with PCOM.

Ovarian morphological assessment via gray-scale ultrasound is shown in Figure 2, highlighting characteristic PCOM features in one of the study participants.

**Figure 3:**
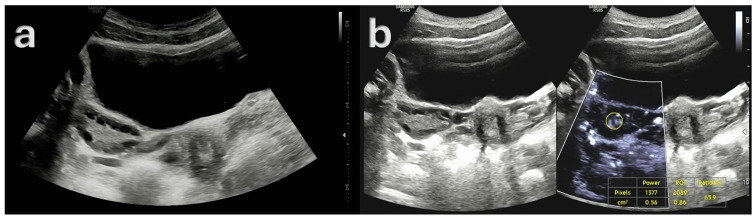
Ultrasound findings in a 23-year-old female patient with Type A polycystic ovary syndrome (PCOS) phenotype. (**a**) Gray-scale ultrasound image demonstrating polycystic ovarian morphology (PCOM), with multiple small peripheral follicles and a prominent central stroma. (**b**) MV-Flow Doppler ultrasound image showing increased vascularity within the ovarian stroma, with a high vascularity index (VI), consistent with elevated stromal blood flow (ratio: 65.9%). These imaging features, in conjunction with clinical and biochemical evidence of hyperandrogenism and ovulatory dysfunction, are characteristic of the classic Type A PCOS phenotype.

Subsequently, Figure 3 presents a quantitative example of ovarian stromal vascularity assessment via MV-Flow Doppler imaging in a woman with PCOM.

The mean vascularity index (VI) was 43.88 ± 19.84. Between the two radiologists, the interobserver agreement for the vascular index measurement was good (ICC = 0.79 (95% CI: 0.65, 0.88)). The distribution of PCOS phenotypes in the cohort was 45.1% for Phenotype A, 22.5% for Phenotype C, and 20.7% for Phenotype D. Vascularity index differed significantly across groups (*p* < 0.001), with the highest mean value in Phenotype A (61.36 ± 10.11), followed by Phenotype C (42.57 ± 3.59), Phenotype D (26.47 ± 4.24), and the lowest in Non-PCOS group (9.95 ± 5.44). Total testosterone, reported as median [IQR], was significantly higher in Phenotype A at 2.14 [1.77–2.34] ng/mL (*p* < 0.001) compared to other groups. Phenotype C demonstrated moderate levels at 1.55 [1.41–1.62] ng/mL, and Phenotype D showed 1.11 [1.10–1.30] ng/mL. The Non-PCOS group had the lowest median total testosterone, 1.00 [0.97–1.06] ng/mL. Free testosterone levels followed a similar pattern (*p* < 0.001). Phenotype A displayed the highest value at 5.00 [3.90–5.37] pg/mL, followed by Phenotype C at 3.20 [2.60–3.90] pg/mL, and Phenotype D at 1.61 [1.51–1.90] pg/mL. The Non-PCOS group measured 1.61 [1.53–1.64] pg/mL. DHEAS levels also differed significantly among PCOS subtypes (p < 0.001). Phenotype D exhibited the highest median of 362 [299–429] μg/dL, followed by Phenotype C at 299 [263–311] μg/dL and Phenotype A at 241 [147–290] μg/dL. The Non-PCOS group, at 152 [136–165] μg/dL, showed the lowest DHEAS levels. Median estradiol levels were 36.01 [28.51–47.00] pg/mL in Phenotype A, 32.75 [22.30–37.70] pg/mL in Phenotype C, 31.82 [29.07–39.36] pg/mL in Phenotype D, and 28.22 [21.23–40.03] pg/mL in the Non-PCOS group. The differences among the groups were not statistically significant (*p* = 0.155). Finally, the LH/FSH ratio (median [IQR]) was significantly different among groups (*p* = 0.002). Phenotype A displayed the highest ratio at 1.34 [0.96–2.12], whereas Phenotype C had the lowest at 0.88 [0.62–1.10]. The Non-PCOS group (0.93 [0.78–1.13]) and Phenotype D (1.10 [0.60–1.51]) exhibited intermediate values.

Descriptive statistics for the study variables and PCOS phenotypes are presented in Table 1.

A statistically significant, very weak negative correlation was observed between VI and FSH (r = *−*0.149, *p* = 0.050), whereas VI exhibited a weak positive correlation with the LH/FSH ratio (r = 0.225, *p* = 0.003) and estradiol (r = 0.178, *p* = 0.019). Notably, strong positive correlations were identified between VI and both total testosterone (r = 0.797, *p* < 0.001) and free testosterone (r = 0.778, *p* < 0.001). In contrast, a moderate negative correlation was observed between VI and DHEAS levels (r = *−*0.483, *p* < 0.001). Correlation coefficients between VI and hormonal parameters are summarized in Table 2.

## 4. Discussion

This study demonstrates a significant association between ovarian stromal vascularization and androgen levels in women exhibiting polycystic ovarian morphology (PCOM), with the highest vascularization indices (VI) observed in Phenotype A, followed by Phenotype C and D, and the lowest values in Non-PCOS group. Strong positive correlations between total and free testosterone levels and VI observed in this study further support the association between ovarian stromal vascularization and hyperandrogenism in the pathogenesis of PCOS [7,20]. Moreover, a moderate inverse correlation between VI and DHEAS was identified, with the highest DHEAS concentrations found in Phenotype D. This suggests that, in this subgroup, the clinical features of PCOS may be predominantly driven by adrenal-derived androgens rather than ovarian stromal vascular mechanisms [21], reinforcing the phenotypic heterogeneity of the syndrome. In addition to these correlations, a more detailed examination of the hormonal profiles across PCOS phenotypes reveals distinct endocrine signatures. Phenotype A demonstrated the most pronounced hormonal alterations, with elevated total and free testosterone levels and the highest LH/FSH ratio, consistent with the classical anovulatory hyperandrogenic PCOS pattern. Phenotype C also exhibited increased androgen levels, although to a lesser extent, and had the lowest LH/FSH ratio among the PCOS phenotypes, aligning with its ovulatory nature. Phenotype D, in contrast, presented with lower total and free testosterone levels but the highest DHEAS concentrations, suggesting a predominant adrenal contribution to androgen excess in this group [21]. While FSH levels were slightly higher in Phenotypes C and D, estradiol levels, although numerically higher in Phenotype A, did not differ significantly between groups (*p* = 0.155), likely reflecting consistent early follicular phase timing of sampling. Emerging literature highlights the role of accelerated neoangiogenesis in PCOS, characterized by increased vascular endothelial growth factor (VEGF) expression, potentially driven by hyperandrogenism and low-grade chronic inflammation [4]. These vascular alterations have been implicated in the pathogenesis of metabolic and reproductive dysfunction, as enhanced ovarian blood flow may further exacerbate androgen excess, anovulation, and infertility [4].

**Figure 4:**
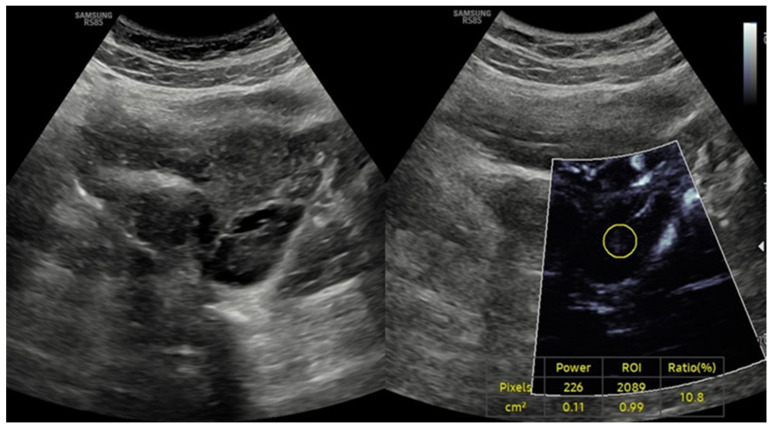
Ultrasound findings in a 26-year-old female patient with isolated polycystic ovarian morphology (PCOM) in the absence of polycystic ovary syndrome (PCOS). Combined gray-scale and MV-Flow Doppler ultrasound images showing multiple small peripheral follicles and a prominent central stroma, consistent with PCOM. The vascularity index (VI), measured on MV-Flow Doppler, is low (ratio: 10.8%), indicating reduced stromal blood flow. The absence of clinical and biochemical hyperandrogenism in this patient supports a diagnosis of isolated PCOM without meeting full diagnostic criteria for PCOS.

The current findings are supported by previous studies employing advanced Doppler techniques to evaluate ovarian vascularization in PCOS. Garg et al. utilized three-dimensional power Doppler ultrasonography to assess ovarian blood flow parameters and reported significantly higher vascularization index (VI), flow index (FI), and vascularization flow index (VFI) in PCOS patients compared to healthy controls, mirroring the increased VI values observed in our hyperandrogenic phenotypes [22]. However, that study did not distinguish between phenotypic subgroups nor assess associations with hormonal parameters, limiting its interpretative depth. Similarly, Senyuva et al. investigated ovarian stromal vascularity using superb microvascular imaging (SMI) and concluded that microvascular techniques may offer greater sensitivity than conventional Doppler methods in detecting vascular alterations in PCOS [19]. Although their findings broadly support increased vascularity in PCOS, the study lacked quantitative analysis and did not provide phenotype-specific correlations. Conversely, Ng et al. reported no significant differences in ovarian stromal blood flow indices between PCOS and control subjects using Doppler ultrasound [23]. Such discrepancies likely reflect methodological heterogeneity across studies, including differences in imaging protocols, ultrasound technologies, and participant selection criteria. These observations are consistent with reports that PCOS is associated with increased ovarian stromal vascularity, particularly in hyperandrogenic phenotypes (Phenotypes A and C) [19,24,25]. Elevated VI values in these phenotypes are consistent with previous studies utilizing color Doppler and three-dimensional ultrasound techniques, which demonstrated increased ovarian blood flow secondary to neoangiogenesis and altered VEGF expression [7,26]. Additionally, a comparative study of ovarian stromal blood flow between PCOS patients and healthy controls revealed significantly lower impedance to flow in the PCOS group, further supporting the presence of enhanced ovarian angiogenesis in this population [27,28]. Notably, polycystic ovarian morphology (PCOM) is frequently observed not only in PCOS patients but also in healthy individuals without endocrine abnormalities (Figure 4).

Previous studies have reported a prevalence of polycystic ovarian morphology (PCOM) ranging from 21% to 63% depending on ethnicity, geographic location, and evolving imaging criteria [29]. In our cohort, PCOM was identified in 11.7% of healthy participants. This overlap underscores the limited specificity of PCOM and highlights the added value of vascular assessment in distinguishing incidental findings from true pathological manifestations of PCOS.

This study has several limitations. The retrospective and single-center design inherently limits the generalizability of the findings. The exclusion of patients on hormonal therapies, while methodologically justified, may have introduced selection bias by omitting individuals with more severe clinical manifestations. Moreover, Phenotype B was not represented in this cohort due to the absence of PCOM, which precluded its inclusion via ultrasound criteria. This is a notable limitation, as it hinders a complete comparative analysis across all PCOS phenotypes. Future studies should include Phenotype B and employ prospective, multicenter methodologies to validate these findings and provide a more comprehensive understanding of the relationship between vascularization and hormonal patterns in PCOS.

In conclusion, our findings highlight the potential utility of microvascular imaging techniques in enhancing the diagnostic accuracy and pathophysiological understanding of PCOS. The observed associations between stromal vascularity and androgen profiles underscore the relevance of vascular alterations, particularly in hyperandrogenic phenotypes. Integration of vascular indices into diagnostic protocols may aid in distinguishing pathological PCOM from incidental findings in healthy individuals, thereby minimizing diagnostic ambiguity and improving individualized clinical management. These findings support the integration of vascular imaging into clinical protocols for PCOS evaluation, though further validation in larger, prospective cohorts remains essential.

## Figures and Tables

**Table 1:** Descriptive statistics and comparison of the study according to PCOS phenotype.

Variable	Total (*n* = 111)	Non-PCOS (*n* = 13)	Phenotype A (*n* = 50)	Phenotype C (*n* = 25)	Phenotype D (*n* = 23)	*p*-Value
Age (years), median (IQR)	23 (21–26)	22 (21–24)	23 (21–26)	24 (21–27)	24 (22–26)	0.665
Mean VI, mean ± SD	43.88 ± 19.84	9.95 ± 5.44 ^a^	61.35 ± 10.11 ^b^	42.57 ± 3.58 ^c^	26.47 ± 4.24 ^d^	<0.001
FSH (mIU/mL), median (IQR)	5.21 (4.13–6.45)	5.37 (4.35–6.02) ^ab^	4.35 (3.35–5.72) ^a^	5.65 (5.00–7.70) ^b^	5.99 (5.54–6.45) ^b^	0.002
LH (mIU/mL), median (IQR)	5.1 (4.00–7.98)	4.7 (4–5.9)	5.5 (4.33–8.61)	5.00 (2.95–6.97)	4.72 (3.79–6.88)	0.307
LH/FSH Ratio, median (IQR)	1.03 (0.75–1.57)	0.93 (0.78–1.13) ^ab^	1.34 (0.96–2.12) ^a^	0.88 (0.62–1.10) ^b^	1.10 (0.6–1.51) ^ab^	0.002
Estradiol (pg/mL), median (IQR)	32.75 (25.31–42.38)	28.22 (21.23–40.03)	36.01 (28.51–47)	32.75 (22.30–37.70)	31.82 (29.07–39.36)	0.155
Total testosterone (ng/mL), median (IQR)	1.62 (1.11–2.06)	1.00 (0.97–1.06) ^a^	2.14 (1.77–2.34) ^b^	1.55 (1.41–1.62) ^c^	1.11 (1.1–1.3) ^ac^	<0.001
Free testosterone (pg/mL), median (IQR)	3.9 (1.9–5)	1.61 (1.53–1.64) ^a^	5.00 (3.9–5.37) ^b^	3.20 (2.60–3.90) ^c^	1.61 (1.51–1.9) ^a^	<0.001
DHEAS (μg/dL), median (IQR)	299 (290–346)	152 (136–165)	241 (147–290) ^a^	299 (263–311) ^b^	362 (299–429) ^b^	<0.001

^a–d^ There is no difference between groups with the same letter. VI = vascularity index FSH = follicle-stimulating hormone LH = luteinizing hormone DHEAS = dehydroepiandrosterone sulfate.

**Table 2:** Correlation coefficients between vascular index and hormonal parameters.

Hormones	Correlation Coefficient (R)	*p*-Value
FSH	–0.149	0.050
LH	0.130	0.090
LH/FSH	0.225	0.003
Estradiol	0.178	0.019
Total Testosterone	0.797	<0.001
Free Testosterone	0.778	<0.001
DHEAS	–0.483	<0.001

FSH: follicle-stimulating hormone; LH: luteinizing hormone; DHEAS: dehydroepiandrosterone Sulfate.

## Data Availability

The data supporting the findings of this study are available from the corresponding author (H.B.) upon request.

## Abbreviations

The following abbreviations are used in this manuscript:

ANOVA	Analysis of variance
CA1-7A	Convex array transducer (1–7 MHz)
CI	Confidence interval
DHEAS	Dehydroepiandrosterone sulfate
FSH	Follicle-stimulating hormone
ICC	Intraclass correlation coefficient
IRB	Institutional Review Board
IQR	Interquartile range
LH	Luteinizing hormone
MVI	Microvascular imaging
MV-Flow	Microvascular flow Doppler
PCOM	Polycystic ovarian morphology
PCOS	Polycystic ovary syndrome
PRF	Pulse repetition frequency
ROI	Region of interest
SD	Standard deviation
SMI	Superb microvascular imaging
US	Ultrasonography
VEGF	Vascular endothelial growth factor
VI	Vascularity Index
SMI	Superb microvascular imaging

## References

[1] Ehrmann D.A. (2005). Polycystic ovary syndrome. N. Engl. J. Med..

[2] Makled A.K., El Sherbiny M., Elkabarity R. (2014). Assessment of ovarian stromal blood flow after metformin treatment in women with polycystic ovary syndrome. Arch. Gynecol. Obstet..

[3] Christ J.P., Cedars M.I. (2023). Current Guidelines for Diagnosing PCOS. Diagnostics.

[4] Kinnear H.M., Tomaszewski C.E., Chang A.L., Moravek M.B., Xu M., Padmanabhan V., Shikanov A. (2020). The ovarian stroma as a new frontier. Reproduction.

[5] Singh S., Pal N., Shubham S., Sarma D.K., Verma V., Marotta F., Kumar M. (2023). Polycystic ovary syndrome: Etiology, current management, and future therapeutics. J. Clin. Med..

[6] Lizneva D., Suturina L., Walker W., Brakta S., Gavrilova-Jordan L., Azziz R. (2016). Criteria, prevalence, and phenotypes of polycystic ovary syndrome. Fertil. Steril..

[7] Emekçi Özay Ö., Özay A.C., Gün İ. (2022). Comparison of stromal thickness and doppler findings in polycystic ovary syndrome and healthy women with ultrasonographic evidence of polycystic ovaries? A cross-sectional study. J. Obstet. Gynaecol..

[8] Ramírez Martín N., Romeu M., Martínez J., Peinado I., Chico-Sordo L., Buigues A., Soriano M.J., Pellicer N., Pellicer A., Herraiz S. (2023). P-647 Underlying mechanisms in clinical manifestations of polycystic ovarian morphology (PCOM) and PCOS women. Hum. Reprod..

[9] Azziz R., Sanchez L.A., Knochenhauer E.S., Moran C., Lazenby J., Stephens K.C., Taylor K., Boots L.R. (2004). Androgen Excess in Women: Experience with Over 1000 Consecutive Patients. J. Clin. Endocrinol. Metab..

[10] Rosenfield R.L., Ehrmann D.A. (2016). The Pathogenesis of Polycystic Ovary Syndrome (PCOS): The Hypothesis of PCOS as Functional Ovarian Hyperandrogenism Revisited. Endocr. Rev..

[11] Abbott D.H., Vepraskas S.H., Horton T.H., Terasawa E., Levine J.E. (2018). Accelerated Episodic Luteinizing Hormone Release Accompanies Blunted Progesterone Regulation in PCOS-like Female Rhesus Monkeys (*Macaca mulatta*) Exposed to Testosterone during Early-to-Mid Gestation. Neuroendocrinology.

[12] Fedeli V., Unfer V., Dinicola S., Laganà A.S., Canipari R., Monti N., Querqui A., Galante E., Laurenzi G., Bizzarri M. (2024). Inositol Restores Appropriate Steroidogenesis in PCOS Ovaries Both In Vitro and In Vivo Experimental Mouse Models. Cells.

[13] Mohamed S.M., Nivya P.S., Devika C.S., Reddy M.S., Poornima N., Roopesh K.R., Kolageri S. (2023). A review of hyperandrogenism state in polycystic ovarian syndrome. Int. J. Reprod. Contracept. Obstet. Gynecol..

[14] Xiang Y., Wang H., Ding H., Xu T., Liu X., Huang Z., Wu H., Ge H. (2023). Hyperandrogenism drives ovarian inflammation and pyroptosis: A possible pathogenesis of PCOS follicular dysplasia. Int. Immunopharmacol..

[15] Agrawal R., Conway G., Sladkevicius P., Tan S.L., Engmann L., Payne N., Bekir J., Campbell S., Jacobs H. (1999). Serum Vascular Endothelial Growth Factor and Doppler Blood Flow Velocities in In Vitro Fertilization: Relevance to Ovarian Hyperstimulation Syndrome and Polycystic Ovaries. Obstet. Gynecol. Surv..

[16] Agrawal R., Sladkevicius P., Engmann L., Conway G.S., Payne N.N., Bekis J., Tan S.L., Campbell S., Jacobs H.S. (1998). Serum vascular endothelial growth factor concentrations and ovarian stromal blood flow are increased in women with polycystic ovaries. Hum. Reprod..

[17] Guzmán A., Hernández-Coronado C.G., Gutiérrez C.G., Rosales-Torres A.M. (2023). The vascular endothelial growth factor (VEGF) system as a key regulator of ovarian follicle angiogenesis and growth. Mol. Reprod. Dev..

[18] Liu M.M., Chen X.H., Lu X.M., Wang F.F., Wang C., Liu Y., Li P.L., Du B.T., Liang S., Gong P.D. (2020). Variations in the Profiles of Vascular-Related Factors Among Different Sub-Types of Polycystic Ovarian Syndrome in Northern China. Front. Endocrinol..

[19] Senyuva I., Turan C.O., Yuksel G.Y., Senturk S. (2023). Superb Microvascular Imaging Doppler Technique in the Evaluation of Ovarian Stromal Vascularity in Women with Polycystic Ovary Syndrome. J. Pak. Med. Assoc..

[20] Fulghesu A.M., Angioni S., Frau E., Belosi C., Apa R., Mioni R., Xamin N., Capobianco G.P., Dessole S., Fruzzetti F. (2007). Ultrasound in polycystic ovary syndrome—The measuring of ovarian stroma and relationship with circulating androgens: Results of a multicentric study. Hum. Reprod..

[21] Yildiz B.O., Azziz R. (2007). The adrenal and polycystic ovary syndrome. Rev. Endocr. Metab. Disord..

[22] Garg N., Khaira H.K., Kaur M., Sinha S. (2018). A Comparative Study on Quantitative Assessment of Blood Flow and Vascularization in Polycystic Ovary Syndrome Patients and Normal Women Using Three-Dimensional Power Doppler Ultrasonography. J. Obstet. Gynaecol. India.

[23] Ng E.H., Chan C.C., Yeung W.S., Ho P.C. (2005). Comparison of ovarian stromal blood flow between fertile women with normal ovaries and infertile women with polycystic ovary syndrome. Hum. Reprod..

[24] Alcázar J.L., Kudla M.J. (2012). Ovarian stromal vessels assessed by spatiotemporal image correlation–high definition flow in women with polycystic ovary syndrome: A case–control study. Ultrasound Obstet. Gynecol..

[25] Battaglia C., Battaglia B., Morotti E., Paradisi R., Zanetti I., Meriggiola M.C., Venturoli S. (2012). Two- and Three-Dimensional Sonographic and Color Doppler Techniques for Diagnosis of Polycystic Ovary Syndrome. J. Ultrasound Med..

[26] Wang W.-Q., Chu G.-H., Hou X.-X. (2023). A comparison of Doppler measures of ovarian blood flow between women with and without ovarian dysfunction and correlations of Doppler indices with ovarian dysfunction markers: A meta-analysis. Ann. Transl. Med..

[27] Dolz M., Osborne N.G., Blanes J., Raga F., Abad-Velasco L., Villalobos A., Pellicer A., Bonilla-Musoles F. (1999). Polycystic ovarian syndrome: Assessment with color Doppler angiography and three-dimensional ultrasonography. J. Ultrasound Med..

[28] Omar Hassan P., Faruq Jamal A. (2024). Intra-ovarian Doppler artery indices values in cases with polycystic ovarian syndrome: A case control study. Zanco J. Med. Sci..

[29] Catteau-Jonard S., Bancquart J., Poncelet E., Lefebvre-Maunoury C., Robin G., Dewailly D. (2012). Polycystic ovaries at ultrasound: Normal variant or silent polycystic ovary syndrome?. Ultrasound Obstet. Gynecol..

